# No impact of transgenic *cry1Ie* maize on the diversity, abundance and composition of soil fauna in a 2-year field trial

**DOI:** 10.1038/s41598-019-46851-z

**Published:** 2019-07-17

**Authors:** Chunmiao Fan, Fengci Wu, Jinye Dong, Baifeng Wang, Junqi Yin, Xinyuan Song

**Affiliations:** 1 Jilin Provincial Key Laboratory of Agricultural Biotechnology, Jilin Academy of Agricultural Sciences, Changchun, 130033 China; 2 Jilin Agricultural University, Changchun, 130118 China

**Keywords:** Environmental economics, Biotechnology

## Abstract

Soil fauna play an essential role in the soil ecosystem, but they may be influenced by insecticidal Cry proteins derived from *Bacillus thuringiensis* (Bt) maize. In this study, a 2-year field trial was conducted to study the effects of transgenic cry1Ie maize, a type of Bt maize (Event IE09S034), on soil fauna, with the near-isogenic line non-Bt maize (Zong 31) as a control. The soil animals were collected with Macfadyen heat extractor and hand-sorting methods, respectively, and their diversity, abundance and community composition were calculated. Then, the effects of maize type, year, sampling time and soil environmental factors on the soil fauna were evaluated by repeated-measures ANOVA, redundancy analysis (RDA) and nonmetric multidimensional scaling (nMDS). Repeated-measures ANOVA showed that the diversity and abundance of the soil fauna were not affected by maize type, while they were significantly influenced by year and sampling time. Furthermore, for both the Macfadyen and hand-sorting methods, RDA indicated that soil fauna community composition was not correlated with maize type (Bt and non-Bt maize) but was significantly correlated with year, sampling time and root biomass. In addition, it was significantly related to soil pH according to the hand-sorting method. nMDS indicated that soil fauna community composition was significantly correlated with year and sampling time; however, it was not associated with maize type. In this study, we collected soil faunal samples according to the Macfadyen and hand-sorting methods and processed the obtained data with ANOVA, RDA, and nMDS in three ways, and our data indicate that transgenic *cry1Ie* maize (Event IE09S034) had no substantial influence on the diversity, abundance or community composition of the soil fauna.

## Introduction

Since the first commercial genetically modified (GM) crop was cultivated in the United States in 1996^1^, GM crops have been planted for 22 years^2–4^. In 2017, the total global planting area reached 189.8 million hectares^5^. With the spread of GM crops, environmental safety problems have caused wide public concern.

At present, the main method for researching the impacts of GM crops on environmental safety is to monitor biodiversity^6,7^ in the field. With this method, many studies have been conducted on the diversity of aboveground species communities^8–12^, but there has been less focus on belowground species communities. Even among the studies related to the belowground species community, most of them focused on soil microorganisms^13–18^. Recently, transgenic *cry1Ab* gene crops, a type of commercialized GM crop, have been studied and showed no impacts on soil organisms^19–29^. For example, transgenic *cry1Ab* rice had no significant effects on the residual decay- and decomposition-associated microbial community compositions in comparison to the non-Bt rice variety (Xiushui 11)^16^. Two types of *cry1Ac*/*cpti* transgenic rice (GM1 and GM2) also had no effects on the composition and abundance of bacterial and fungal communities in paddy soil during the growing season^15^. Bt hybrid cotton MECH-162 did not have adverse effects on both culturable and nonculturable microbial diversity according to the analysis of microbial community structure dynamics^18^. Mohammad *et al*. (2003) and Toschki *et al*. (2007) reported that both Bt maize and transgenic *cry1Ab* maize had no adverse effects on soil faunaus^19,21^. To date, no detrimental impacts of GM crops on belowground organisms have been found.

The Institute of Plant Protection, Chinese Academy of Agricultural Sciences (CAAS), isolated and cloned the anti-insect gene *cry1Ie*^30^, which encodes an insecticidal protein that does not show cross resistance with several other insecticidal proteins, Cry1Ab, Cry1Ac, Cry1Ah and Cry1F^25,26,31,32^, and shows certain virulence to the Asian corn borer (ACB) and cotton bollworm^33^. Therefore, the *cry1Ie* gene can be integrated with Bt genes, such as *cry1Ah*, to form stacked transgenic insect-resistant maize and thus to overcome the problems resulting from the high homology and interactive resistance of the single Bt gene maize type^27,34^. At present, the transgenic *cry1Ie* maize hybrid (Bt maize, Event IE09S034) is being tested for commercial production in China and has the potential to be commercialized.

Some studies have revealed that transgenic *cry1Ie* maize has no impact on the diversity of arthropod communities in the field^35–37^, while no study on the belowground soil fauna community has been reported until now.

In this study, we conducted field trials to compare the effects of transgenic *cry1Ie* (Bt maize, IE09S034) and non-Bt (Zong 31) maize hybrids on soil fauna, and the soil fauna were sampled according to two methods (the Macfadyen and hand-sorting methods). Then, we used the diversity and abundance parameters RDA and nMDS to analyse the soil fauna community and to evaluate the effects of the transgenic *cry1Ie* maize hybrid on soil fauna in the field. This study provides useful data for the commercialization of GM crops in the future.

## Results

### Soil fauna in Bt and non-Bt maize plots

**Figure 1:**
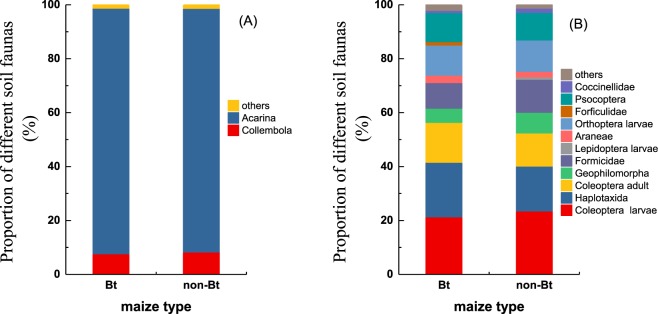
The taxon percentages of soil fauna found in Bt and non-Bt maize plots in 2-year field trials. Macfadyen method in 2014 and 2015 (**A**) hand-sorting method in 2014 and 2015. (**B**) This diagram illustrates every dominant soil fauna taxon with a proportion >1% and a total proportion of taxa <1% (others).

When the soil fauna community was extracted according to the Macfadyen method, in 2014 and 2015, a total of 20,133 and 21,777 soil animals were found in the Bt and non-Bt maize plots, respectively, and the animals in the two types of maize field both belonged to 11 species. In both years, the animals of Acarina accounted for the majority of the soil fauna in both Bt and non-Bt maize fields. The percentage of Acarina species was more than that of other species, which was 91.09 and 90.39% in the Bt and non-Bt maize fields, respectively, followed by Collembola species, which represented 7.59 and 8.25% of the animals in the Bt and non-Bt maize fields, respectively (Fig. 1A) and (Table 1).

**Table 1:** The abundance of soil animals captured in Bt and non- Bt maize fields by two years in Macfadyen and hand-sorting methods respectively.

Method	Class	Order	Family	Bt	non-Bt
Macfadyen	Entognatha	Collembola		1528	1796
	Arachnida	Acarina		18339	19685
	Insecta	Diplura		22	19
	Insecta	Coleoptera larvae		8	4
	Insecta	Psocoptera		53	49
	Insecta	Hemiptera	Formicidae	16	17
	Insecta	Coleoptera	Staphylinidae	1	2
	Insecta	Coleoptera adult		14	12
	Clitellata	Enchytraeidae		40	47
	Clitellata	Haplotaxida		101	144
	Chilopoda	Lithobiomorpha		0	2
	Chilopoda	Geophilomorpha		11	0
Hand-sorting	Clitellata	Haplotaxida		96	80
	Chilopoda	Lithobiomorpha		2	2
	Chilopoda	Geophilomorpha		25	37
	Insecta	Coleoptera larvae		101	113
	Insecta	Coleoptera adult		70	59
	Insecta	Hemiptera	Formicidae	45	59
	Insecta	Lepidoptera larvae		0	6
	Insecta	Araneae		13	10
	Insecta	Orthoptera	Gryllidae	2	0
	Insecta	Orthoptera larvae		53	56
	Insecta	Hemiptera		4	0
	Insecta	Dermaptera	Forficulidae	6	4
	Insecta	Psocoptera		51	49
	Insecta	Coleoptera	Coccinellidae	6	8

When analysed according to the hand-sorting method, 474 and 481 soil animals were found in the Bt and non-Bt maize fields in 2014 and 2015, respectively. There were 13 and 12 species found in the Bt and non-Bt maize fields, respectively. In addition, the percentage of animals in each phylum was analysed. Coleoptera larvae, Haplotaxida, Coleoptera adults, Orthoptera larvae, and Psocoptera in the Bt maize plots accounted for 21.31, 20.25, 14.77, 11.18, and 10.76%, respectively, and the percentages of Coleoptera larvae, Haplotaxida, Coleoptera adults, Orthoptera larvae, Formicidae, and Psocoptera species in the non-Bt maize plots were 23.94, 16.63, 12.27, 11.64, 12.27, and 7.59%, respectively (Fig. 1B) and (Table 1).

### Impacts of maize type, year and sampling time on soil fauna diversity and abundance

Repeated-measures ANOVA results showed that maize type had no significant effect on the Shannon-Wiener diversity index (*H*′), Simpson’s diversity index (*D*), Pielou’s evenness index (*J*), the number of species (*S*), or the total animal number (*N*) according to the Macfadyen and hand-sorting methods, indicating that transgenic *cry1Ie* maize did not influence the soil fauna. In addition, ANOVA showed that year, sampling time, and their interactions all had significant impacts on the *H*′, *D*, *J*, *S* and *N* of the soil fauna in the two types of maize plots when investigated according to the Macfadyen method, which indicated that the soil fauna was affected by year and sampling time. No effects of the interactions of year × maize type and year × maize type × sampling time on *H*′, *D*, *J*, *S* and *N* of soil fauna were detected for either method (Table 2).

**Table 2:** Effects of year (2014 and 2015), maize type (Bt maize and non-Bt maize) and sampling time on soil fauna diversity and abundance, analyzed using a three-way unequally spaced repeated-measure ANOVA.

Method	Variable	Diversity and abundance parameter									
		Shannon-Wiener index		Simpson’s diversity index		Pielou’s evenness index		Number of species		Abundance	
		(*H*′)		(*D*)		(*J*)		(*S*)		(*N*)	
		*F*	*P*	*F*	*P*	*F*	*P*	*F*	*P*	*F*	*P*
Macfadyen	Year	13.37	**0**.006^**^	22.59	**0**.001^***^	23.97	**0**.001^***^	10.13	**0**.013^*^	9.00	**0**.017^*^
	Maize type	0.12	0.739	0.05	0.829	0.02	0.907	2.00	0.195	0.07	0.803
	Sampling time	4.69	**0**.004^**^	2.88	**0**.038^*^	3.91	**0**.011^*^	4.10	**0**.009^**^	15.41	**0**.003^**^
	Year × Maize type	1.04	0.339	0.97	0.354	0.43	0.531	0.50	0.500	0.06	0.82
	Year × Sampling time	5.30	**0**.002^**^	5.27	**0**.002^**^	6.15	**0**.001^***^	3.27	**0**.020^*^	19.43	**0**.002^**^
	Maize type × Sampling time	0.49	0.743	0.36	0.834	0.80	0.536	0.31	0.867	0.06	0.831
	Year × Maize type × Sampling time	0.86	0.500	0.68	0.614	0.40	0.806	0.57	0.683	0.03	0.892
	Mean ± SD (Bt maize)	0.847 ± 0.131		0.325 ± 0.056		0.391 ± 0.060		4.833 ± 0.464		671.100 ± 159.636	
	Mean ± SD (non-Bt maize) maize)	0.818 ± 0.190		0.317 ± 0.083		0.387 ± 0.084		4.567 ± 0.654		725.900 ± 238.092	
Hand-sorting	Year	0.06	0.821	0.20	0.668	0.09	0.768	0.04	0.850	0.58	0.468
	Maize type	0.53	0.487	0.88	0.377	0.87	0.379	0.04	0.850	0.35	0.573
	Sampling time	1.07	0.387	0.60	0.667	0.37	0.828	1.80	0.153	1.62	0.193
	Year × Maize type	0.35	0.572	0.11	0.753	0.00	0.96	1.37	0.275	0.58	0.468
	Year × Sampling time	3.16	**0**.027^*^	3.54	**0**.017^*^	3.17	**0**.026^*^	1.74	0.166	3.11	**0**.029^*^
	Maize type × Sampling time	2.25	0.085	1.25	0.312	0.64	0.637	2.24	0.086	1.52	0.221
	Year × Maize type × Sampling time	1.02	0.411	0.96	0.441	0.95	0.450	0.45	0.771	0.89	0.482
	Mean ± SD (Bt maize)	2.046 ± 0.172		0.734 ± 0.061		0.828 ± 0.058		5.700 ± 0.504		17.000 ± 2.562	
	Mean ± SD (non-Bt maize)	2.142 ± 0.211		0.770 ± 0.052		0.858 ± 0.040		5.767 ± 0.733		16.100 ± 2.595	

The values highlighted in bold are statistically significant (*P < 0.05; **P < 0.01; ***P < 0.001).

Repeated-measures ANOVA also showed that the root biomass (*P* = 0.258), soil water content (*P* = 0.2888) and soil pH (*P* = 0.067) in the Bt and non-Bt maize fields were not significantly different, indicating that the Bt and non-Bt maize had similar amounts of root biomass and that they had no significant effect on soil environmental factors, that is, soil water content and soil pH.

### Effects of maize type and soil environment factors on soil fauna

**Figure 2:**
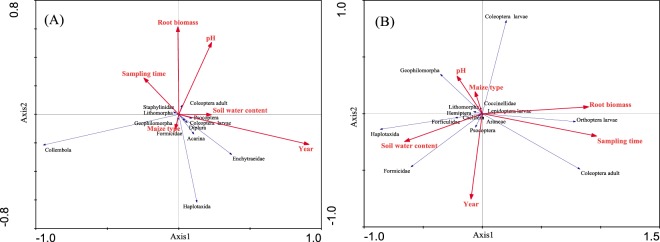
Redundancy analysis (RDA) of the relationships among soil fauna community compositions and between soil fauna community compositions and environmental factors. (**A**) The soil fauna were collected according to the Macfadyen method; (**B**) the soil fauna were collected according to the hand-sorting method.

According to the Macfadyen and hand-sorting methods, year, sampling time and soil environmental variables together explained 25 and 32% of the total variability in the community composition of soil fauna collected according to the Macfadyen and hand-sorting methods, respectively, while maize type was not related to this variability (Table 3). For the Macfadyen method, axes 1 and 2 explained 49.7 and 75.0% of the variation in the species-environment relationship, respectively, with eigenvalues of 0.125 and 0.064 and species-environment correlations of 0.726 and 0.514, respectively (Fig. 2A). For the hand-sorting method, axes 1 and 2 explained 59.9 and 89.4% of variation in the species-environment relationship, respectively, with eigenvalues of 0.190 and 0.094 and species-environment correlations of 0.805 and 0.721, respectively (Fig. 2B).

**Table 3:** Effects of maize type, sampling time, year and soil environment factors on the soil animals investigated according to the Macfadyen and hand-sorting methods in a Monte Carlo test of a RDA.

Environmental factor	Macfadyen			Hand-sorting		
	Variance explained (%)	*F*	*P*	Variance explained (%)	*F*	*P*
Year	11	7.17	**0**.0020^**^	18	12.86	**0**.0020^**^
Sampling time	5	3.46	**0**.0100^**^	6	4.27	**0**.0020^**^
Root biomass	5	3.44	**0**.0140^*^	4	2.84	**0**.0080^**^
pH	3	2.02	0.0660	3	2.17	**0**.0260^*^
Soil water content	1	0.75	0.5700	1	0.52	0.8200
Maize type	0	0.32	0.9140	0	0.61	0.7500
Total	25			32		

The values highlighted in bold are statistically significant (*P < 0.05; **P < 0.01).

The relationship between soil fauna and maize type or other factors (year, sampling time, maize root biomass, soil water content and soil pH) was explored with the RDA method. The Monte Carlo test shows that when soil animal samples were collected according to the Macfadyen method, year (*F* = 7.17, *P* = 0.0020), sampling time (*F* = 3.46, *P* = 0.0100) and root biomass (*F* = 3.44, *P* = 0.0140) were significantly correlated with the composition of soil fauna. This result may have occurred because the different years and sampling times were associated with different soil temperatures and soil water contents, which influence the growth and development of soil fauna. In addition, root biomass is not only used as a type of food but also produces some substances that influence soil pH and the soil environment, so it also impacts the composition of soil fauna. When analysing the data from the hand-sorting method according to RDA, maize type did not impact the soil fauna, and soil water content (*F* = 0.52, *P* = 0.8200) was not significantly correlated with the composition of soil fauna. The soil fauna was highly related to soil pH (*F* = 2.17, *P* = 0.0260) when it was investigated according to the hand-sorting method, which was different from the result obtained according to the Macfadyen method. This may be because the hand-sorting method allows the capture of more soil animal species than the Macfadyen method (Table 3).

According to the Macfadyen method, the soil fauna of Collembola, Haplotaxida and Enchytraeidae were more easily affected by year, sampling time, maize root biomass, soil water content and pH than other soil animals. Among them, the number of Collembola was positively correlated with sampling time. This was due to Collembola reproduction increasing with increasing temperature. However, the number of Haplotaxida and Enchytraeidae were negatively correlated with sampling time, which might be due to changes in the soil water content (Fig. 2A). According to the hand-sorting method, the soil fauna of Coleoptera larvae, Geophilomorpha, Haplotaxida, Formicidae, Coleoptera and Orthoptera larvae were more easily affected by year, sampling time and environmental factors than other animals. Among them, Geophilomorpha, Haplotaxida, Formicidae, and Orthoptera larvae were all significantly negatively correlated with sampling time, while Coleoptera was significantly positively correlated with sampling time, which might have occurred because they have different suitable soil temperatures (Fig. 2B).

### The similarity of soil fauna communities in Bt and non-Bt maize fields

**Figure 3:**
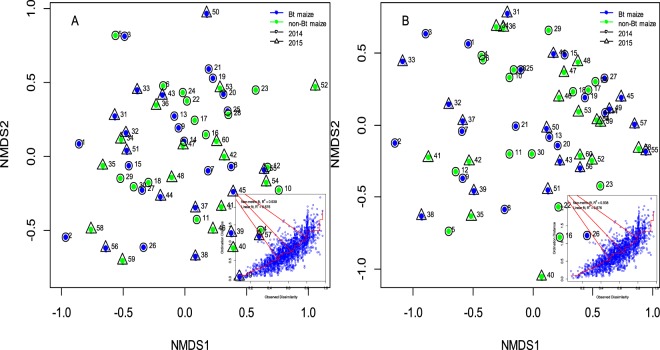
Non-metric multidimensional scaling (nMDS) plot of soil fauna community structure in different maize fields. Macfadyen method (**A**) and hand-sorting method (**B**) *Circles* with associated numbers from 1 to 30 indicate sampling points analysed in temporal order in 2014 (1–3: Bt maize at V0 stage, 4–6: non-Bt maize at V0 stage, 7–9: Bt maize at V3 stage, 10–12: non-Bt maize at V3 stage, 13–15: Bt maize at V6 stage, 16–18: non-Bt maize at V6 stage, 19–21: Bt maize at R1 stage, 22–24: non-Bt maize at R1 stage, 25–27: Bt maize at R6 stage, 28–30: non-Bt maize at R6 stage). *Triangles* with associated numbers from 31 to 60 indicate sampling points analysed in temporal order in 2015 (31–33: Bt maize at V0 stage, 34–36: non-Bt maize at V0 stage, 37–39: Bt maize at V3 stage, 40–42: non-Bt maize at V3 stage, 43–45: Bt maize at V6 stage, 46–48: non-Bt maize at V6 stage, 49–51: Bt maize at R1 stage, 52–54: non-Bt maize at R1 stage, 55–57: Bt maize at R6 stage, 58–60: non-Bt maize at R6 stage). The right side of the diagram represents Shepard’s stress plot.

The soil fauna community structures in the Bt and non-Bt maize fields were further explored by nMDS. The distance between two sampling points was estimated using the pairwise Bray-Curtis similarity index. As shown in Fig. 3, the differences in the soil fauna composition among all animal samples were visualized with the nMDS plot, in which the samples were separated according to sampling time and year but not by maize type (Fig. 3A,B), which was substantiated by the more detailed analysis of similarity (ANOSIM). Significant correlations between soil fauna community composition and year or sampling time were found; however, no correlation between maize type and community composition was detected for soil fauna collected according to the Macfadyen and hand-sorting methods (Table 4).

**Table 4:** Effects of maize type (Bt and non-Bt maize), sampling time and year on soil fauna community structure analysed according to the nMDS method.

Correlation with nMDS structure	Macfadyen		Hand-sorting	
	*R* ^2^	*P*	*R* ^2^	*P*
Sampling time	0.29	**0**.001^***^	0.35	**0**.001^***^
Year	0.01	**0**.016^*^	0.00	**0**.013^*^
Maize type	0.00	0.953	0.00	0.601

The values highlighted in bold are statistically significant (*P < 0.05; ***P < 0.001).

## Discussion

Transgenic crops can cause the retention of foreign gene expression products in soil through crop stubble, root exudates and pollen transmission^38–41^ and may cause changes in soil composition and content near the plant rhizosphere, which in turn affect the abundance and diversity of the soil fauna, ultimately posing a potential threat to the multiple functions of soil ecosystems^42–44^. At present, most related studies focus on the impacts on plants and surface animals, and only some specific orders, such as Collembola^45–48^, Haplotaxida^28,29,49^ and Enchytraeidae^19,20,23^. However, the overall structure and function of the soil fauna in the field are comprehensive.

In this study, soil fauna were investigated according to the Macfadyen and hand-sorting methods^50–52^. The combination of the two methods can comprehensively reflect changes in soil fauna. The two methods were successfully used to investigate the effects of different fertilization treatments on the soil fauna in the black soil area of Jilin Province, China^52^. In addition, the Shannon-Wiener index, Simpson’s index, Pielou’s evenness index, number of species and abundance have been successfully used to study the effects of transgenic crops on the arthropod community in the field^35,36^. Therefore, we used these five parameters to study the effects of transgenic *cry1Ie* maize IE09S034 on soil fauna. In this study, we analysed and compared the diversity of soil fauna in transgenic *cry1Ie* insect-resistant and non-transgenic control maize fields and concluded that the soil fauna were not significantly affected by the maize materials.

In recent years, RDA and nMDS analysis methods have been used for many types of ecological studies and evaluating the impact of transgenic crop cultivation on animal communities in the field^53,54^. This study also used the two methods to analyse the relationship between maize type and soil fauna composition and showed that transgenic maize did not have a significant impact on soil fauna.

Recently, as collembolans are a dominant animal group in crop fields and are sensitive to environmental changes^55,56^, they have been used as an indicator for evaluating the environmental safety of transgenic crop cultivation. For example, Mina^45^ *et al*. explored whether transgenic Bt cotton Mech162 endangered environmental safety by analysing its effects on the population structure of collembolans. Heckmann^46^ *et al*. found that transgenic Bt maize had no significant inhibitory effect on *Protaphorura*, and they concluded that the cultivation of Bt maize was not harmful to the environment. In addition, Chang^47^ *et al*. and Zhu^48^ *et al*. found that transgenic crops influenced the abundance of collembolans and concluded that the cultivation of transgenic crops was not safe for the environment. However, the effects of environmental factors on soil collembolans were not considered in the above studies. Collembolans are a dominant soil animal group, so they can be used as another indicator of the environmental safety of transgenic Bt maize. We considered the effects of environmental factors on collembolans through RDA and concluded that it is the environmental conditions (i.e., maize root biomass) but not maize type (transgenic or non-transgenic maize) that significantly affects the soil collembolan community.

According to the hand-sorting method, Haplotaxida, as a dominant group, was found to be an excellent index of the soil ecological environment due to its advantages in terms of promoting soil organic matter circulation and improving soil structure and fertility^49^. Some studies have shown that Haplotaxida can promote the decomposition of Bt proteins in soil and have no adverse effects on Haplotaxida themselves^49^. Zeilinger^28^ *et al*. only used four species of Haplotaxida to indicate whether transgenic *cry1Ab* and *cry3Bb1* maize had effects on soil fauna. However, with the exception of the studied factor, the above studies related to Haplotaxida did not consider environmental effects. In this study, we concluded that Bt maize had no significant influence on the Haplotaxida community, which is more credible for the study considering the effects of environmental factors, such as maize root biomass and soil water content, identified through RDA.

As nMDS can be used to analyse the similarity of soil community structures^57,58^, Guo^35,36^ *et al*. used this method to study the effect of transgenic *cry1Ie* maize on the arthropod community in the field. In this study, we also used the nMDS ordination method to analyse the differences in soil fauna between transgenic and non-transgenic maize fields and illustrated that transgenic maize did not significantly affect the soil fauna^58^. This result was consistent with the RDA results. In this study, we used two sampling methods (Macfadyen and hand-sorting) and two analytical methods (RDA and nMDS) to explore whether transgenic *cry1Ie* maize (IE09S034) harmed the environment and concluded that the cultivation of transgenic *cry1Ie* insect-resistant maize did not influence the soil ecology. This result supports the popularization of transgenic *cry1Ie* maize (IE09S034) in spring maize production areas in China in the future.

## Materials and Methods

### Ethics statement

Field experiments were conducted using transgenic *cry1Ie* corn IE09S034 (insect resistant) in Jilin Province from 2013 to 2016, which was approved by the Ministry of Agriculture and Rural Affairs of China. In this study, no vertebrates were included, and none of the species are endangered or protected.

### Materials and experimental design

#### Experimental materials

Bt maize (transgenic *cry1Ie* maize hybrid (IE09S034)) and near-isogenic non-Bt maize (Zong 31) were used in the study, which were provided by the Institute of Crop Sciences, CAAS. Field trials were conducted in 2014 and 2015 at the National Centre for Transgenic Plants Research and Commercialization/Jilin Academy of Agricultural Sciences in Gongzhuling (43°30′N, 124°49′E), Jilin Province, China. The soil at the experimental field is the typical black soil of northeast China, which contains 27.08 ± 0.07 g · kg^−1^ of organic matter, 77.54 ± 0.07 mg · kg^−1^ of alkaline nitrogen, 10.68 ± 0.07 mg · kg^−1^ of available phosphorus, and 154.10 ± 0.76 mg · kg^−1^ of available potassium. Before our experiments, only the non-GM hybrid Zhengdan 958 had been planted for the last three years. We sowed maize seeds on 7 May in 2014 and on 6 May in 2015. A randomized block design with three blocks was employed. Each plot was 10 m wide and 15 m long and contained 25 rows with 60-cm spacing. There were 40 plants in one row that were 25 cm apart. All plots were separated by 2 m bare borders. Both seeding and weeding were manually performed, and no chemical pesticides were applied during the entire growing period. Except for the lack of insecticide application, the maize was cultivated using the same agricultural management practices as normal.

#### Soil animal sampling

Macfadyen^59^ and hand-sorting^60,61^ methods were used to collect soil fauna before sowing (V0) and during four maize growing stages of the 3^rd^ leaf (V3), elongation (V6), silking (R1), and physiological maturity (R6) stages in both years.

For the Macfadyen method, each time, we selected five sampling points from which to extract soil fauna from each plot. The five points were randomly selected every time in each plot. At each point, we collected soil samples from around the maize roots using a soil auger (15 cm in diameter, 10 cm in height). The soil samples were mixed, and 200 mL was collected with a measuring cylinder. Soil was collected within the maize row between two corn plants and approximately 12 cm away from each of the two plants. Each time, we took 30 soil samples in total from three replicates of the two maize types. The 200 mL sample was placed on a mesh with a mesh size of 20 on a Macfadyen extractor funnel, and the soil invertebrates were extracted for seven days at room temperature (25 °C). The soil invertebrates moved down through the funnel and dropped into a collection bottle below with 95% alcohol. The number and species of the collected animals were analysed at a magnification of 40 times with a microscope (Motic, China). Different soil animals were identified according to the keys of Yin^62^, Zhong^63^ and Zhang^64^. The numbers of soil animals collected five times at the five sites in one plot were summed for analysis.

In addition, we removed 5 g of soil from the 200 mL soil sample and dried it in an oven at 105 °C for the calculation of the actual water content.

Furthermore, 5 g of soil was mixed with an equal volume of 0.01 mol/L CaCl_2_, shaken for 5 minutes, and maintained under constant conditions for 2–24 h, and then the soil pH value was measured with a pH metre (Dynamica, UK).

To analyse the soil fauna in every plot with the hand-sorting method, only three of the above five sampling sites were randomly used because the sampling sites used in this method occupy a relatively large area and at each site two maize plants would be totally damaged each time. At each hand-sorting experimental site, we dug out a block of soil (0.5 m length × 0.5 m width × 0.5 m height) around the maize roots. The soil fauna were counted by the naked eye and collected with tweezers, the process of which was finished within 15 minutes. The total number of soil fauna in each plot was used as the soil fauna number per plot. The collected soil fauna were also stored in 95% ethanol for species identification and analysis. All roots of six maize plants in a plot destroyed by the hand-sorting method were collected, dried, weighed, and used as maize root biomass (g) per plot.

### Statistical analysis

The changes in fauna occurrence, abundance, and diversity were analysed with the Data Processing System (DPS) version 2005 package (China)^65^. Three indices, the Shannon-Wiener index (*H*′), Simpson’s diversity index (*D*), and Pielou’s evenness index (*J*), were calculated as follows:

$$H^{\prime} =-\,\sum _{i=1}^{s}{P}_{i}\,\mathrm{ln}\,{(P}_{i})$$

where *P*_*i*_ is the proportion of individuals belonging to the *i*^*th*^ taxon in one plot^66^.

$$D=1-\,\sum _{i=1}^{s}\frac{{N}_{i}(Ni-1)}{N(N-1)}$$

where *N*_*i*_ is the number of individuals in the *i*^*th*^ taxon in one plot and *N* is the total number of individuals in one plot^67^.

$$J=H^{\prime} /\,\mathrm{ln}\,S$$

where *S* is the number of faunal genera collected from one plot.

These three diversity indices were calculated for the Macfadyen and hand-sorting methods in 2014 and 2015 using the diversity index analysis module in the DPS.

Repeated-measures ANOVA (SPSS 23.0)^68^ was used to analyse the effects of every factor on soil fauna abundance and diversity, including maize type (Bt and non-Bt maize), year and sampling time. We also used repeated-measures ANOVA to analyse whether the environmental factors (maize root biomass, soil water content and soil pH) between Bt maize and non-Bt maize were different. For the above analysis, maize type (Bt and non-Bt maize), year and sampling time were included as fixed factors, and block and sampling points were considered as random factors.

A type of canonical analysis, redundancy analysis (RDA), was used to identify the factors influencing the soil fauna community by analysing the relationships among soil fauna and the relationships between the soil fauna and the soil environment^69^ and can be performed with Canoco and CanoDraw (Microcomputer Power, USA). Monte Carlo permutation tests (499 permutations) were performed to test the significance of the canonical axes of the RDA^70,71^. Additionally, an indirect ordination method, nonmetric multidimensional scaling (nMDS), was used to identify the effects of every influencing factor on the soil fauna community, which illustrated the similarity of the soil fauna samples through metric multidimensional scale analysis using the Bray-Curtis distance between sampling points^57,58^, and^69^. Analysis of similarity (ANOSIM) was used to calculate the distance between two sampling points according to the Bray-Curtis algorithm, and then all distances were sorted from small to large^58^. This analysis was conducted with the vegan package in R ver.3.2.3 (Auckland University, New Zealand)^72–75^.
